# Asymmetrical activation and asymmetrical weakness as two different mechanisms of adolescent idiopathic scoliosis

**DOI:** 10.1038/s41598-021-96882-8

**Published:** 2021-09-02

**Authors:** Yulhyun Park, Jin Young Ko, Joon Young Jang, Seungeun Lee, Jaewon Beom, Ju Seok Ryu

**Affiliations:** 1 Department of Rehabilitation Medicine, Seoul National University Bundang Hospital, 82 Gumi-ro 173 Beon-gil, Bundang-gu, Seongnam-si, Gyeonggi-do 463-707 South Korea; 2 Department of Rehabilitation Medicine, Seoul National University College of Medicine, Seoul, South Korea

**Keywords:** Medical research, Diseases, Neurological disorders

## Abstract

There have been many studies on adolescent idiopathic scoliosis related abnormal muscle contractions of the spine. However, previous studies using surface electromyography to investigate paraspinal muscle asymmetry are controversial, lacking in clarity of results, and hindered by methodological limitations. The purpose of this study was to investigate the relationship between imbalance factors including surface electromyography activity according to the scoliosis curve type and leg length discrepancy and adolescent idiopathic scoliosis curve types. Seventy-nine patients with scoliosis were prospectively enrolled and were divided into five types: single thoracic, thoracolumbar, lumbar, double thoracic, and double major. Cobb angle and structural variables were measured. Surface electromyography examinations were conducted at the 7th, 12th thoracic erector spinae, 3rd lumbar erector spinae, and multifidus muscles during the superman position keeping prone spinal extension to lift the arms and legs off the floor. Whole spine radiographs were obtained to measure the Cobb angle, coronal imbalance, pelvic height and angle, and femoral head height. In the double major, thoracolumbar, and lumbar types, the mean root mean squared (RMS) EMG amplitudes were significantly higher on the convex side than the concave side (*P* < 0.005). In the DM type, the mean RMS EMG amplitudes of ES_T7_ and ES_L3_ where the apex was located were significantly higher at the convex side than those of the concave side (*P* < 0.005, effect size (Cohen’s d) for ES_T7_/ES_L3_: 0.517/0.573). The TL and L types showed a similar pattern. The mean RMS EMG amplitudes of the ES_T12_ concave side and Mu_L3_ and ES_L3_ concave sides were significantly lower than those of the convex side in the TL and L types, respectively (*P* < 0.008, effect size (Cohen’s d) for ES_T12_/Mu_L3_/ES_L3_: 0.960/0.264/0.448). Conversely, there were no significant differences in the single thoracic and double thoracic types. All structural variables (coronal imbalance, pelvic height and angle, and femur head height) were higher in the lumbar type, but only coronal imbalance was significantly different (*P* < 0.05). Different patterns of asymmetry of paraspinal muscles and structural variables were described based on the curvature of the spine. L type showed that EMG activity was asymmetric in the paraspinalis muscles where the apex was located and that structural asymmetry, such as coronal imbalance was significantly greater than other types. DM type showed similar paraspinalis asymmetry pattern to the ST type but there was no structural asymmetry in DM and ST types. TL type has the features of both thoracic and lumbar origins. Understanding these could contribute to the management in correcting scoliosis.

## Introduction

Scoliosis is defined as a lateral curvature > 10° of the spine on a standing coronal radiograph and a three-dimensional deformity of the spine^1^. A majority (approximately 80%) of scoliosis cases have no definite etiology and are referred to as idiopathic scoliosis (IS). Adolescent idiopathic scoliosis (AIS) is the most common form of IS^2^. The etiology of this condition is probably multifactorial; many theories exist, based on different principles^3^.

Several possible causes have been suggested. Asymmetry of the paraspinal muscles was thought to be one of the causes; there have been various studies on paraspinal muscle asymmetry using surface electromyography (S-EMG). Studies have reported that S-EMG activity was found to be higher on the convex side of the scoliotic curve; these findings suggest an overactivation of the paraspinal muscles as a cause of AIS^4–7^. Conversely, de Oliveira et al. reported that there was no significant difference in electromyographic amplitude of erector spinae on the convex and concave sides^8^. These conflicting results might be due to methodological differences, based on patient selection, arbitrary focus on specific curve types or lack of controlling mechanisms to exclude inadequate posture^7,9–11^. Since these studies describe conflicting results, our research attempted disaggregate analysis of the relationship between paraspinal muscle asymmetry and scoliosis curve types, considering how the curve classifications and recording levels were not accurately described in previous studies.

Timgren et al. investigated the association between leg length discrepancy (LLD), pelvic asymmetry, and scoliosis based on clinical examination results, and concluded that asymmetrical postural balance was correlated with convexity and concavity of the spinal curve^12^. Though there is a proven link between scoliosis and LLD, the prior study only compared the locations of the curve and the LLD and did not analyze LLD according to the curve types and Cobb angle.

The etiology of scoliosis might be multifactorial including paraspinal asymmetry and LLD, so we explored the factors which contribute to Cobb angle and differences between curve types. Analysis of these parameters based on curve types could provide more information regarding our understanding of AIS. Subsequently, this information could be used to develop more precise, science-based exercise treatment. Therefore, the primary purpose of this study was to discover the difference among paraspinal muscles imbalance, asymmetrical structural variables (LLD and coronal imbalance), and the AIS curve types. The secondary purposes were to identify what factors are most consistently associated with Cobb angle and search for factors with high correlation according to the scoliosis types.

## Methods

This study was a single-center prospective cohort study. All patients or their guardians, if the patients were under 18, provided informed consent. The study protocol was approved by the Institutional Review Board of Seoul National University Bundang Hospital (B-1701/378-103). The study protocol was registered on Clinicaltrials.gov under reference no. NCT03497520 (Initial release: 13/04/2018, Actual study start date: 17/02/2017, Actual study completion date: 21/02/2020, Last release: 22/07/2020). All methods were carried out in accordance with the Declaration of Helsinki principles^13^.

### Volunteers

AIS patients referred by clinics or orthopedic surgeons were assessed by our department and screened accordingly. A screening program was carried out on 101 patients with scoliosis by conducting Adam’s forward bending test for spinal rotation deformity or rib hump and by taking whole spine radiograph. Participants were enrolled prospectively from February 2017 to February 2020. The inclusion criteria were patients over nine years and under 19 years old, diagnosis of AIS, and radiographic examination. Patients were excluded if Cobb angle < 10°, they had any pre-existing orthopedic or systemic condition, any history of central nervous system diseases, neuromuscular diseases, and congenital spinal abnormalities.

### Radiographic assessment

**Figure 1:**
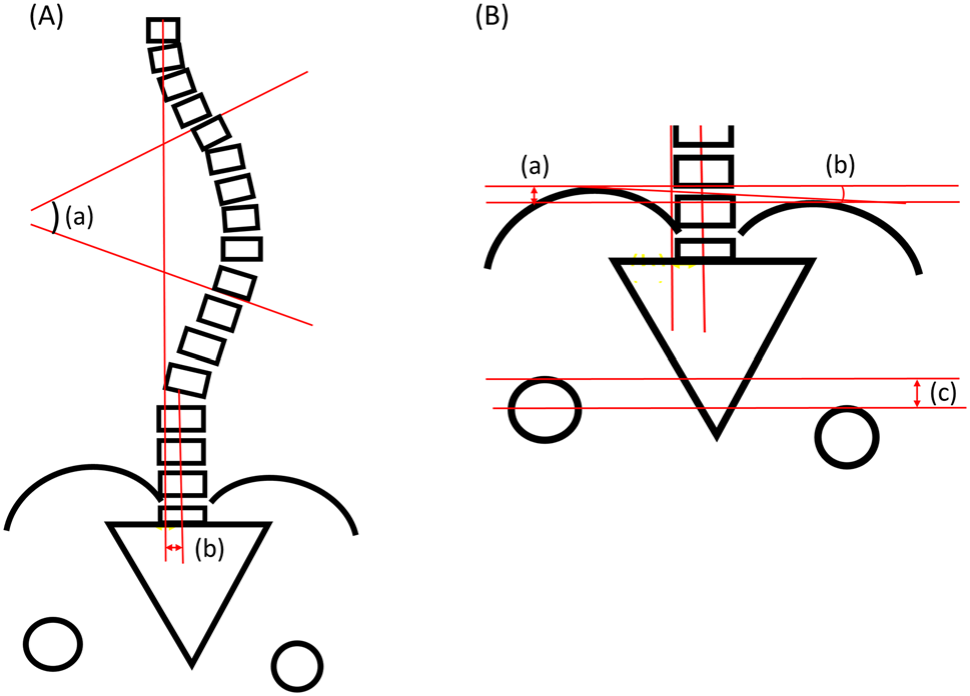
Figure (**A**) shows how we measured the Cobb angle (a) and coronal imbalance (b). Coronal imbalance was defined as the horizontal distance between the C7 plumb line and the center sacral vertical line in coronal plane. Figure (**B**) shows how we measured pelvic height (a) and angle (b), and femoral head height (c). Pelvic height and angle were defined as the vertical distance and angle between bilateral upper border of iliac crest. Femur head height was defined as the vertical distance between the upper borders of both femur heads.

Anteroposterior whole spine or EOS radiographs were obtained with the patient in the standing position. These images were used to measure the Cobb angle, coronal imbalance, pelvic height and angle, and femoral head height. Coronal imbalance was defined as the horizontal distance between the C7 plumb line and the center sacral vertical line in the coronal plane. Pelvic height and angle were defined as the vertical distance and angle between the upper borders of both iliac crests, respectively. Femur head height was defined as the vertical distance between the upper borders of both femur heads (Fig. 1). Pelvic height and femoral head height on standing PA radiographs or EOS radiographs are a simple and reliable method for LLD documentation with an average magnification of 4.6%^14,15^.

**Figure 2:**
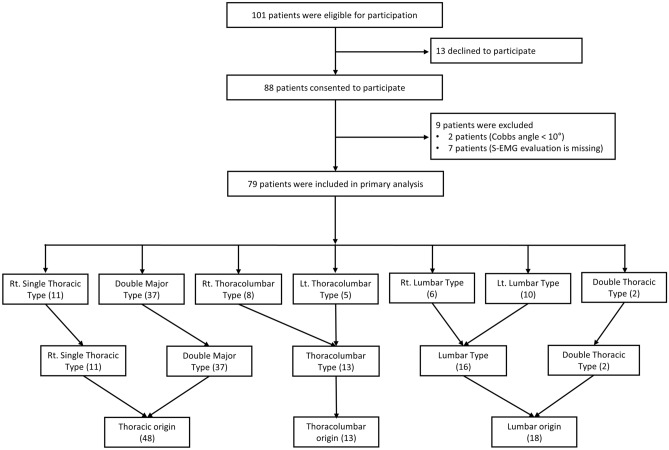
This figure shows the algorithm for study enrollment and classification of the patients.

Curve types in this study were modified on the basis of the Lenke classification and Scoliosis Research Society-Schwab Classification Definitions^16^. To evaluate curve types, only the coronal profile was used. The participants (n = 79) were divided into five types: Rt. Single thoracic (ST, n = 11), a single main thoracic curve with the apex between the T2 and T11; Thoracolumbar (TL, n = 13), a major thoracolumbar curve with the apex at T12-L1; Lumbar (L, n = 16), a major lumbar curve with the apex between L2 to L4; Double thoracic (DT, n = 2), major main thoracic and minor proximal thoracic curves, and Double major (DM, n = 37), structural thoracic and lumbar curves. There was no triple major curve type (Fig. 2). The demographic data of the subjects including age, sex, height, weight, and Cobb angle were obtained; these details are provided in Table 1.

**Table 1:** Demographic characteristics of scoliosis patients according to curve types.

Curve type (N)	Male (%)	Age (yrs.)	Weight (kg)	Height (cm)	BMI	VAS	SRS22 SUM	SRS22 Mean	Surgery (%)	Brace (%)*	Cobbs angle (°)		
											Thoracic	TL	Lumbar
Total (79)	15.0	13.6 ± 2.3	45.7 ± 11.0	156.7 ± 11.5	18.5 ± 2.7	1.8 ± 2.3	42.9 ± 12.7	2.0 ± 0.6	0	28.8			
Single thoracic (11)	9.1	13.2 ± 2.8	40.5 ± 11.3	151.2 ± 12.8	17.3 ± 2.8	1.8 ± 1.7	39.7 ± 11.8	1.8 ± 0.5	0	27.3	25.9 (11.4–44.3)		
Thoracolumbar (13)	26.7	13.4 ± 2.7	48.5 ± 14.6	155.7 ± 12.0	19.6 ± 2.8	2.2 ± 2.7	41.1 ± 12.5	1.8 ± 0.7	0	6.7		19.0 (10.1–29.5)	
Lumbar (16)	18.8	14.0 ± 2.3	46.7 ± 10.6	158.3 ± 13.5	18.6 ± 2.9	2.1 ± 2.9	46.4 ± 13.1	2.1 ± 0.6	0	18.8			22.7 (10.1–37.1)
Double major (37)	10.8	13.6 ± 2.1	45.7 ± 9.4	157.6 ± 9.9	18.3 ± 2.5	1.5 ± 2.0	43.4 ± 13.1	1.8 ± 0.8	0	37.8	24.9 (13.6–46.6)		23.7 (11.2–43.7)
Double thoracic (2)	0	14.5 ± 2.6	48.5 ± 9.2	165.4 ± 1.3	17.8 ± 3.6	2.5 ± 3.5	37.0 ± 5.7	1.7 ± 0.3	0	100	33.8 (23.8–45.2)	31.1 (27.6–36.8	

Values are presented as mean ± SD. *P value < 0.05.

BMI, Body mass index; VAS, Visual Analogue Scale; SRS, Scoliosis Research Scale, TL, Thoracolumbar.

In case of double curve type, the Cobb angle was measured at two levels. The Cobb angle was measured by two independent investigators who had no knowledge of subject status. If the curve types of the two investigators did not match, we selected the one that matched the curve type measured by a third investigator.

### Electromyographic measures

Initially, S-EMG examinations were conducted prospectively in patients with AIS for quantitative analysis of muscle activation patterns. The 7th and 12th thoracic and 3rd lumbar levels represent the common apex of the curve types^17^. Electrodes for the S-EMG were placed bilaterally 2 cm lateral to the spinous process for the 7th and 12th thoracic erector spinae and 3rd lumbar multifidus muscles, and 4 cm lateral for the 3rd lumbar erector spinae muscles (ES_T7_, ES_T12_, Mu_L3_, ES_L3_)^18,19^. To evaluate the degree of muscle contraction during exercise, a wireless S-EMG analysis system (BTS FREEEMG 1000 with EMG-BTS EMG Analyzer, BTS Bioengineering Co.) was used for electrophysiological quantitative analysis. The recording time was 10 s. Then, the root mean square (RMS) was obtained. All subjects were instructed to maintain a specific posture keeping both hands and legs raised with the patient in the prone position (superman position). This posture is a standardized high load postural task for recruitment of spinal extensor muscles. This task was repeated twice, and the average values were analyzed.

As all patients with the ST type were convex to right, only the TL and L types were found to have bilateral convexities. The apex area affected by scoliosis can be described as convex or concave. However, in this study, bilateral sEMG values were obtained even in non-apex areas to investigate asymmetry for each level of the spine. To match bilateral convexities, we turned the statistics by changing the direction of the left and right so that convex side could be unified to the right. Thereby, the TL and L types were unified with respect to the convex to right curve type. The mean RMS values were compared between the convex and concave sides at each paraspinal muscle level to determine the correlation between paraspinal asymmetry and the curve types.

### Reclassification according to the different mechanisms using paraspinalis asymmetry ratio

When we visualized the paraspinalis asymmetry ratio by dividing the types with left and right convex, we found a similarity of asymmetric ratios between ST and DM type, and DT and L, right convex type. To determine the different mechanisms, we reclassified them by origin: thoracic origin (ST, DM types), thoracolumbar origin, and lumbar origin (L, DT types) groups.

### Statistics

All statistical analyses were conducted using the SPSS 23.0 software (SPSS Inc., Chicago, IL, USA). Standard descriptive statistics was used to calculate the normalized RMS means and standard deviations (SD) for both groups. To compare mean RMS values between the convex and concave sides at each paraspinalis muscle level, paired T test or Wilcoxon’s signed ranks test (if the assumptions of parametric statistical analysis were not satisfied) was used at the same level. For correlation analysis, this paraspinalis asymmetry was converted into an asymmetry ratio (right/left) and analyzed. To correct for multiple comparisons, FDR (False Discovery Rate)-Benjamini–Hochberg procedure was used to *P* value correction, so the level of statistical significance was less than 0.0045 to 0.008 for these analyses.

The Kruskal–Wallis test was used to compare the coronal imbalance, pelvic height and angle, and femur head height in the ST, TL, L, DT, and DM types. Spearman’s and Pearson’s correlation coefficients were used to compare Cobb angle and the variables (RMS asymmetry ratio and structural variables). The Kruskal–Wallis test was used to compare the S-EMG values and structural variables among the thoracic origin, thoracolumbar origin, and the lumbar origin groups. The level of statistical significance was less than 0.05 for analyses.

## Results

A total of 101 patients who visited the outpatient clinic for scoliosis underwent screening. Of these, 13 declined to participate, two were excluded because the Cobb angles were < 10°, and seven were excluded in the primary analysis because there was no S-EMG data. Finally, a total of 79 patients were included in the primary analysis (Fig. 2). All patients underwent anteroposterior whole spine radiographs; whole-body EOS radiographs were additionally obtained from 58 patients.

Demographic characteristics are described in Table 1. Median age was 13.6 ± 2.3 years; the mean Cobb angles of each curve group were T: 25.9 ± 13.1°, TL: 19.0 ± 6.3°, L: 22.7 ± 8.1°, DM: 24.9 ± 9.1° (T) and 23.7 ± 8.5° (L), and DT: 33.8 ± 11.7° (T) and 31.1 ± 9.1° (TL). Only the use rate of brace was significantly different among the groups; the brace was most frequently used in the DM type and least in the TL type (Table 1).

**Figure 3:**
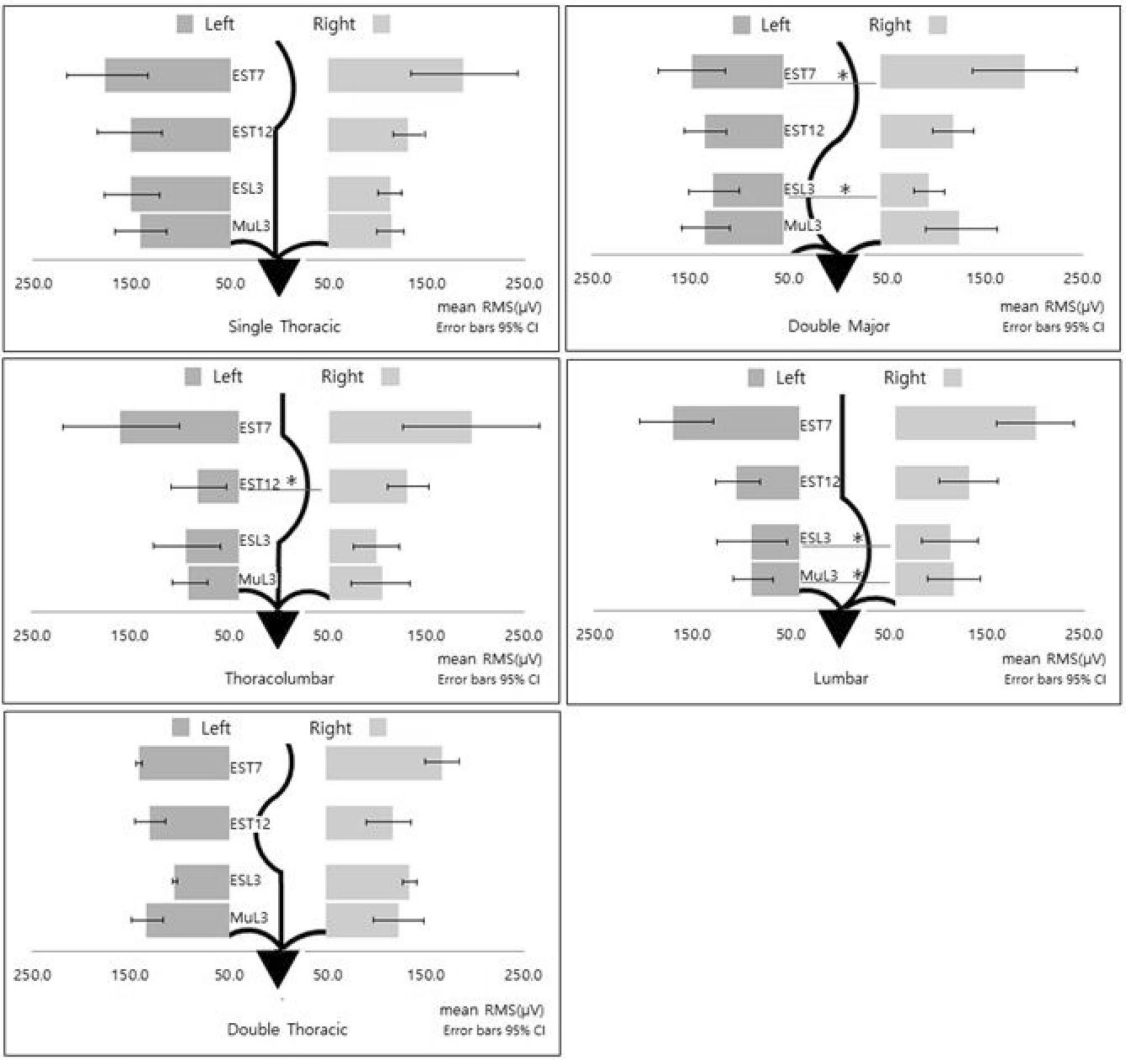
Comparison of the S-EMG mean RMS values of the T7, T12, and L3 paraspinal muscles according to the curve types. Single thoracic, double major, thoracolumbar unified, lumbar unified, and double thoracic types.

The differences of the mean RMS values between the paraspinal muscles on the concave and convex sides are shown in Fig. 3. In the DM type, the mean RMS of the ES_T7_ and ES_L3_ where the apex was located were significantly higher on the convex side than on the concave side (*P* < 0.008). In the ES_T12_ without curve, there was no asymmetry and the convex/concave ratio was 0.99 ± 0.25, which was close to 1.

The TL and L types showed a similar pattern. The mean RMS of the ES_T12_ concave side and Mu_L3_ and ES_L3_ concave sides were significantly lower than those of the convex side in the TL and L types, respectively (*P* < 0.008). There was no asymmetry between sides where the apex was not located. Unlike DM, TL, and L types, there were no significant differences between the convex and concave sides at the apex in the ST and DT types. Although there was no statistically significant difference between sides among the ST and DT types, the mean RMS value of the convex side in the apex area was relatively higher than that of the concave side. When we compared the paraspinalis muscle asymmetry ratio (right/left) among the types, the ES_L3_ ratio in the L type was significantly different from other types (*P* < 0.05).

**Figure 4:**
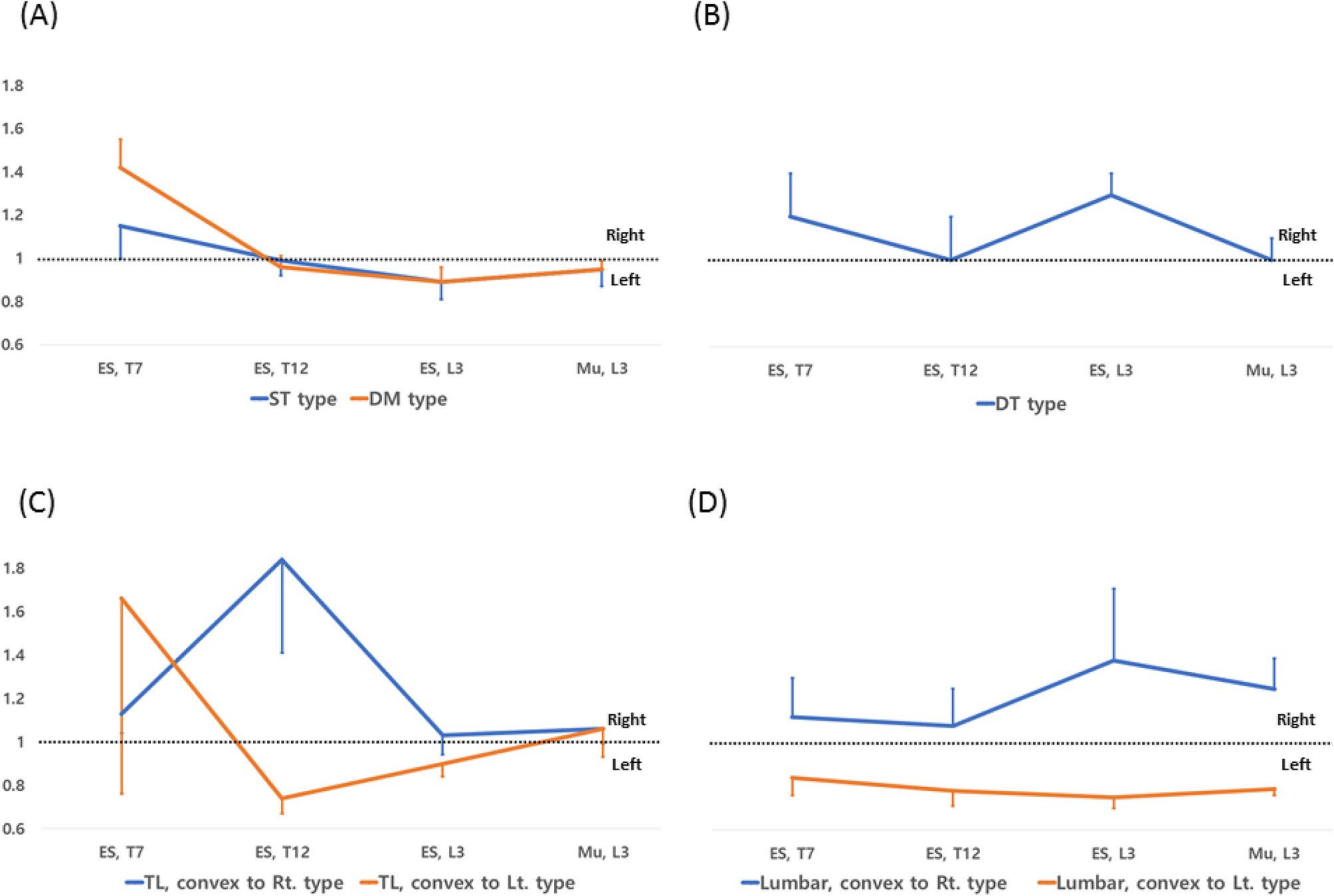
These figures illustrate asymmetry ratio by dividing the types showing apex convexity to the right and left in (**A**) single thoracic (ST) and double major (DM) types, (**B**) Double thoracic (DT) type, (**C**) thoracolumbar (TL), convex to right or left types, and (**D**) lumbar, convex to right or left types. In these figures, the asymmetric ratio showed similarity between ST and DM type, and between DT and lumbar, convex to right type. In addition, the asymmetric ratios of bilateral directions were opposite at the T12 level, and at all levels (T7, T12, and L3 levels) in TL and lumbar types, respectively. The asymmetric ratio at T7 level was positive regardless of curve direction in TL type.

The convex/concave asymmetry ratio of the paraspinal muscles by curve type is illustrated in Fig. 4, showing respective apex convexity sidedness in each group. ST and DM types showed a convex/concave ratio greater than one in ES_T7_ and ES_L3_. In ES_T12_ and Mu_L3_, the convex/concave ratio was approximately one, indicating that both sides were symmetrical. In the case of TL type, the convex/concave ratio appeared according to the direction of the curve apex at ES_T12_, but the ratio in ES_T7_, right side showed the dominant RMS value regardless of the direction of convexities. At the L3 level where the curve was not visible, the convex/concave ratio was close to one. On the other hand, in the case of L type, asymmetry was shown at all paraspinal muscle levels in the direction of the curve apex, and in the case of DT, the pattern was similar to that of the L, right convex type.

Comparisons between curve types and structural variables (coronal imbalance, pelvic height and angle, and femur head height) are described in Table 2. Though all structural variables were higher in the L type, only coronal imbalance was significantly different (*P* < 0.05).

**Table 2:** The structural variables according to the types of scoliosis.

Curve type	N	Coronal imbalance (mm)	Pelvis heights (mm)	Femur head heights (mm)	Pelvis angle degree (°)
Single thoracic	11	7.6 ± 5.7	3.0 ± 3.8	1.2 ± 1.9	1.2 ± 1.4
Thoracolumbar	13	10.9 ± 10.0	3.9 ± 4.4	2.1 ± 2.7	1.5 ± 1.6
Lumbar	16	14.6 ± 8.2	6.7 ± 5.4	4.1 ± 4.2	2.2 ± 1.7
Double major	37	8.0 ± 10.1	4.2 ± 4.4	2.6 ± 3.4	1.5 ± 1.5
Double thoracic	2	15.1 ± 2.6	2.1 ± 3.0	0.0 ± 0.0	0.8 ± 1.1
*P* value		0.020	0.329	0.126	0.421

Values are presented as mean ± SD.

Correlation analysis between the Cobb angle and variables (RMS asymmetry ratio and structural variables) are described in Table 3. The ES_L3_ ratio and MuL3 ratio were strongly correlated with the Cobb angles in the thoracolumbar spine (r = 0.667, *P* = 0.003 and r = 0.531, *P* = 0.040). The lumbar Cobb angle was only moderately correlated with MuL3 ratio (r = 0.332, *P* = 0.017). Other RMS asymmetry ratio and structural variables were not correlated with the degree of Cobb angle in all curve types.

**Table 3:** Correlations between Cobb angle, paraspinalis asymmetry ratio, spinal deviation, and pelvis and hip parameters.

Curve location	ES_T7_ ratio	ES_T12_ ratio	ES_L3_ ratio	Mu_L3_ ratio	Coronal imbalance	Pelvic heights	Femur head heights	Pelvis angle
Thoracic Cobb angle	0.154 (0.271)	− 0.057 (0.686)	0.226 (0.103)	0.249 (0.072)	0.110 (0.431)	0.002 (0.991)	0.170 (0.223)	− 0.024 (0.867)
Thoracolumbar Cobb angle	0.212 (0.397)	0.172 (0.494)	0.667 (0.003)	0.531 (0.023)	0.334 (0.176)	0.210 (0.419)	0.206 (0.427)	0.100 (0.704)
Lumbar Cobb angle	0.245 (0.083)	− 0.073 (0.611)	0.288 (0.040)	0.332 (0.017)	0.056 (0.695)	0.258 (0.068)	0.207 (0.145)	0.217 (0.126)

Values are presented as correlation coefficients (*P* value).

Comparisons of paraspinal asymmetry variables and structural variables after reclassification by origin are presented in Table 4. Based on the data obtained above, comparison was performed for three groups as thoracic (ST and DM), thoracolumbar (TL), and lumbar (L and DT) origin groups according to the types showing a similar convex/concave ratio pattern. When thoracic, thoracolumbar, and lumbar origins were compared, the RMS ratio at ES_T12_ of TL origin were significantly higher than those of the thoracic and lumbar origins. In addition, coronal imbalance was significantly higher in the lumbar origin than other origins.

**Table 4:** The comparisons of muscular and structural variables between the location of origin group.

		Thoracic origin group (ST + DM)	Thoracolumbar origin group (TL)	Lumbar origin group (L + DT)	*P* value
Locations	Number	48	13	18	
Muscular activation variables	T7 ratio	1.4 ± 0.7	1.3 ± 1.2	1.2 ± 0.5	0.475
	T12 ratio	1.0 ± 0.3	1.7 ± 10.0	1.2 ± 0.4	0.000
	L3 ratio	1.2 ± 0.6	1.1 ± 0.2	1.4 ± 0.4	0.091
	L3 Mu ratio	1.1 ± 0.4	1.1 ± 0.2	1.2 ± 0.2	0.077
Structural variables	Coronal imbalance	7.9 ± 9.3	11.0 ± 10.0	14.7 ± 7.7	0.003
	Pelvis heights	4.0 ± 4.2	3.9 ± 4.4	6.2 ± 5.3	0.293
	Femoral head heights	2.3 ± 3.2	2.1 ± 2.7	3.7 ± 4.2	0.334
	Pelvis angle	1.5 ± 1.5	1.5 ± 1.6	2.0 ± 1.7	0.390

Values are presented as mean ± SD.

ST, single thoracic, DM, double major, L, lumbar, DT, double thoracic.

*P < 0.05.

## Discussion

In this study, it was found that the convex side of the paraspinalis muscles have higher RMS values during isometric contraction than the concave side at the apex of spinal curvature. There have been various studies on the asymmetry of the paraspinalis muscles in patients with AIS. Opinions differ considerably regarding using S-EMG to determine paraspinalis muscle activation in patients with AIS. In some studies, the authors showed that the paraspinalis muscles on the concave side have higher EMG activity, while others could not find asymmetrical activity in the paraspinalis muscles of scoliosis patients^8,9,11^. However, recent literature has reported dominance of EMG activity on the convex side of the scoliotic curve^4–7^. In this study, we measured S-EMG in a specific posture (superman position) to induce proper submaximal contraction of the paraspinalis muscles. As scoliosis develops in the upright position, which requires sustained tonic contractions of the paraspinalis muscles, the superman position can reflect physiologic status better, compared to isokinetic movement. In addition, clear asymmetry was confirmed in this study by further disaggregating analysis by scoliosis type. Further, it was confirmed that the asymmetry pattern varies by type. In the case of the ST type, sidedness of the convexity was exclusively rightward, and the DM type also showed the same pattern at thoracic level. We attribute this to a prospective correlation with the high prevalence of right handedness. The similar ratio of about 90% of right-handedness and the left-sidedness of the concavity suggests that excessive use of the right hand is relevant^20^. Previous studies also reported the relationship between handedness and scoliosis configuration in patients with the thoracic curve type^21,22^. However, to the best of our knowledge, such an analysis specifically aimed at evaluating this correlation has not been previously conducted, suggesting that further research is needed.

In fact, ST and DM types showed similar paraspinal muscle asymmetry ratios not only at the thoracic level but also at other levels in this study (Fig. 4). If DM type is a progression of ST type, this may explain why the ES_L3_ RMS asymmetry occurs in the ST type. The above finding also suggests that the ES_L3_ RMS asymmetry is not always a secondary finding when describing the curvature of the spine.

In the case of the TL type, paraspinal muscle asymmetry had a different patter from the ST, DM, and the L types. Though the directions of the apex at T12 are in concordance with the L type, the RMS values of ES_T7_ were high on the right side regardless of the sidedness of the curve. Therefore, it is possible that the TL type shared characteristics of the thoracic origin group (ST and DM types) and lumbar origin group (L, DT types). Correlation analysis also showed that ES_L3_ asymmetry ratio has linear correlation with the Cobb angle of the TL and L types (Table 3).

Previous study showed the signs of dystrophy and atrophy in the back muscles and differences in proportions of type I fibers versus type II fibers with greater of the former on the apex of the convex side^2,7^. Not only the reduced proportion of type I fiber on the concave side but also the decrease in total muscle volume was observed^23^. This asymmetry may be responsible for the development of scoliosis, but this study was cross-sectional study, therefore, we could not suggest causality of scoliosis. Regardless of whether muscle atrophy of the concave side is the cause or the result, it is thought that strengthening the paraspinalis muscles ipsilateral to the concave side balances tension of the back muscles, which is helpful in the treatment of scoliosis. Also, since the asymmetry differs by type and the paraspinal asymmetry also affects the adjacent level where the curve is not visible, confirming the asymmetry pattern through S-EMG and strengthening the back muscle is helpful, considering the curve type and asymmetry pattern.

As one of the important results, all structural variables were higher in the L type and coronal imbalance reached statistically significant level. It is possible that small LLDs cause repetitive stress in activities of everyday life such as walking and running, which affect the occurrence of the L type. The correlation between LLDs and the lumbar curve indicates that LLDs compensate for the lumbar curve or vice versa^14^. So, the lumbar type is might related to both paraspinalis muscle imbalance and structural LLD. Although EOS radiograph reflects true LLD better than whole spine radiograph, this study did not accurately measure leg length, especially when the pelvis was in the state of rotation, so follow-up studies are needed.

There are some limitations to this study. First, there were no comparisons with a control group. Second, we did not measure maximum voluntary isometric contraction (MVIC) and normalization. Normalization of EMGs is necessary because of the many technical, anatomical, physiological, sex, and investigator factors that can influence the magnitude of EMG^24,25^. To normalize the EMG values, a task specific, standardized submaximal task (isometric/dynamic) is needed^26^. The posture we instructed in this study showed a higher peak amplitude than other back extension exercises, and it can be assumed that EMG normalization was corrected through this standardized submaximal task^19^. In the present study, we used the ratios between convex and concave side instead of using MVIC ratio. Third, the S-EMG recording of ES_T7_ reflects the activation of not only the paraspinalis muscles but also the rhomboids and trapezius muscles. The recordings of ES_L3_ reflect not only erector spinae but also iliopsoas and quadratus lumborum. As the muscles with long lever arms have higher deformational forces, follow-up studies evaluating both paraspinalis and large muscles are required. For example, we need to attach electrodes between the scapular and spinous process targeting the rhomboids and middle trapezius. Fourth, this study was a cross-sectional physiologic study so we could not identify any causal relationships. A future prospective longitudinal study to evaluate scoliosis causality is required. Fifth, there are cases where the lumbar curve is caused by LLD and cases where the thoracic curve is less than 10 degrees among the double major curves. In this study, the lumbar type was not classified based on the left–right direction and structural factors such as LLD according to the directions were not included. Future studies evaluating both directions and structural variables are required.

In conclusion, the present study discovered that that the contributions of muscular and structural asymmetries differ by type of scoliosis. The paraspinalis muscles showed asymmetric muscle activation at certain regions where spinal curvature has occurred. Conversely, L type not only showed the EMG activity imbalance where the apex was located, but also the structural asymmetry. Finally, the TL type is thought to have features of both thoracic and lumbar origins.

## Data Availability

All data in this study is available after de-identification upon request.
